# How have Ontario Public Health units engaged with faith-based organizations to build confidence in COVID-19 vaccines among ethno-racial communities

**DOI:** 10.1371/journal.pgph.0003924

**Published:** 2024-12-31

**Authors:** Kadidiatou Kadio, Melodie Yunju Song, Anna Karbasi, Denessia Blake-Hepburn, Shaza A. Fadel, Sara Allin, Anushka Ataullahjan, Erica Di Ruggiero

**Affiliations:** 1 Centre for Global Health, Dalla Lana School of Public Health, University of Toronto, Toronto, Canada; 2 Institut de Recherche en Science de la Santé du Centre National de la Recherche Scientifique et Technologique (IRSS/CNRST), Ouagadougou, Burkina Faso; 3 Centre for Vaccine Preventable Diseases, Dalla Lana School of Public Health, University of Toronto, Toronto, Canada; 4 Clinical Public Health Division, Dalla Lana School of Public Health, University of Toronto, Toronto, Canada; 5 Institute of Health Policy, Management, and Evaluation, Dalla Lana School of Public Health, University of Toronto, Toronto, Canada; 6 North American Observatory on Health Systems and Policy (NAO), Dalla Lana School of Public Health, University of Toronto, Toronto, Canada; 7 Social and Behavioural Health Sciences Division, Dalla Lana School of Public Health, University of Toronto, Toronto, Canada; 8 School of Health Studies, Faculty of Health Sciences, Western University, London, Canada; PLOS: Public Library of Science, UNITED STATES OF AMERICA

## Abstract

In Ontario, collaborations between Public Health Units (PHUs) and faith-based organizations (FBOs) and other community organizations were implemented to deliver interventions aimed at building trust in vaccines among ethnoracial communities. This research sought to explore the processes of PHU engagement with FBOs, and challenges encountered. A qualitative research study based on in-depth interviews was conducted with 18 of the 34 Ontario PHUs who expressed an interest. Braun and Clarke’s "experiential" approach was used to explore the realities of PHUs’ contextual experiences and perspectives. PHUs developed a two-phased process for engaging with FBOs and ethnoracial communities. First, PHUs created internal frameworks for dialogue to use available data to better understand the diverse needs of these equity-seeking groups. The second phase involved a three-stage engagement process:1) Consultation and information sharing was employed to facilitate early and open dialogue. 2) Work with FBOs and interested communities to plan vaccine deployment strategies to meet the needs of different faith and ethno-racial groups, and jointly plan the implementation of vaccination clinics. 3) Share roles and responsibilities with FBOs to roll out vaccine confidence strategies. The PHUs’ openness to honest dialogue with FBOs, commitment to building relationships based on respect for different beliefs and opinions about vaccines, and previous experience working together all facilitated engagement. Lessons learned from this research can guide the implementation of future vaccination programs. Ensuring early and regular engagement with FBOs a priority strategy and devoting substantial resources (human, financial and duration) are both necessary to improve vaccine confidence and promote equity for ethno-racial groups.

## 1. Introduction

The COVID-19 pandemic further highlighted the urgency of vaccination as an essential public health intervention for reducing population health impacts of infections. Yet vaccine hesitancy or the delay or refusal of vaccination [1, 2] is a major obstacle to ensuring equitable distribution of the health and economic benefits of vaccination [3, 4]. Vaccine hesitancy refers to a heterogeneous category of people who share varying degrees and motives of indecision [5]. For example, resistant groups refer to those who may have no interest in receiving the COVID-19 vaccines despite receiving additional information about them. The World Health Organization (WHO) attributes vaccine hesitancy to three factors: complacency, convenience, and confidence [2]. Confidence refers to trust in the effectiveness and safety of vaccines, as well as the system that delivers them [2]. Minoritized ethnoracial populations are ethnic and racial groups whose human rights, values and cultural practices are undermined by majority ethnoracial groups [6]. Those with low confidence in vaccines also have low trust in COVID vaccines [3, 7–9] due to a legacy of systemic racism and medical mistreatment [10–12]. Vaccine confidence has been particularly undermined by historical and contemporary contexts of systemic racism, marginalization and oppression faced by Indigenous, Black and other racialized groups in Canada and other countries [12–15]. Contemporary experiences and views on vaccination must therefore be contextualized against a history of medical abuses targeting Black and Indigenous communities across North America [11]. Ethnoracial groups are often described as hesitant groups or groups that may have been historically disenfranchised by health systems and/or groups that do not have a clear understanding of COVID-19 vaccines including how they are manufactured, their contents and effects. Yet these groups may still be willing to accept vaccination.

Various community engagement strategies and interventions have been devised to bolster vaccine confidence and counter vaccine hesitancy at the population level and among ethnoracial communities. Community engagement is the cornerstone of community-based public health services [16, 17]. In public health, community engagement is defined as actions involving communities in decision-making and in the planning, design, governance, and delivery of services [18, 19]. Engagement covers a spectrum from more passive to active involvement [20]. It can result in providing partners with the necessary information to understand a problem, to obtaining their feedback on options and decisions to be made, to regular interactions throughout the project cycle, and collaborating [18, 20, 21]. It involves shared decision-making with communities to carry out essential health system functions and local and innovative solutions [18, 22]. Community engagement is considered essential for improving health equity among communities made structurally vulnerable because it can influence action on the socio-structural determinants of health [23]. Two perspectives explain why community engagement can improve people’s health, although many models merge the two perspectives, arguing in favor of community involvement for utilitarian and social justice purposes [18, 24]. A "utilitarian" perspective in which engagement is used instrumentally to increase participation and acceptance of interventions considered appropriate to improve service use and outcomes. In this way, we seek to involve communities to improve the effectiveness of the intervention, the content of which is often designed without the community [24]. Peers, for example, are often mobilized in the hope that their knowledge of the context and their empathy and credibility will benefit the effective implementation of interventions [24, 25]. The “social justice” perspective that promotes empowerment and focuses on inequalities. Engagement is seen as a framework for action towards an ideal to be achieved. This perspective also recognizes the heterogeneous nature of communities, and hierarchy, inter- and intra-community power relations [26]. Community empowerment addresses some of the social determinants of health. It promotes social and structural change by helping people to participate, negotiate, influence control, and hold to account the institutions that affect them [24]. It can give a “voice to the voiceless” and is therefore considered invaluable in addressing health inequities [27, 28]. From this perspective, the health system respects community-defined priorities, recognizes their strengths, and challenges, and builds capacity to enable effective community engagement and empowerment [18].

Public health agencies (PHAs) are organizations whose official mandate is to promote public health within a specific jurisdiction. PHAs’ engagement with faith-based organizations (FBOs) can contribute to the achievement of equity objectives, through the collaborative implementation of the principles of inclusion, flexibility, and trust in the community to promote the vaccine confidence [29–32]. FBOs are defined as “entities whose organizational control, expression of religion, and program implementation are tied to values and beliefs belonging to specific religious identities” [33].

The involvement of community-based organizations such as FBOs is paramount, as these community groups possess the cultural competence and relational capital necessary for fostering open dialogues about vaccines [34]. Notably, interventions to increase vaccine confidence are more effective when tailored to the cultural nuances of ethnic-racial groups [35]. Among these interventions, those involving FBOs have shown promise due to their potential for leveraging trusting and existing networks and communication channels [35, 36]. Evidence syntheses indicate that FBO-engaged interventions contribute to increased vaccine acceptance rates within the ethnoracial communities [36, 37]. For example, one study involving three racialized groups (Black, Indigenous, Latino) found that using testimonials from local leaders and elected officials who have received the COVID-19 vaccine is an effective way to increase confidence in COVID-19 vaccines [7]. Successful community engagement is underpinned by shared objectives, respectful engagement, and effective communication channels [38]. However, challenges such as cultural dissonance and resource disparities must also be navigated [39].

In Canada, PHAs consider community engagement a core competency in public health [40]. The Ontario Public Health Standards include health equity as a foundational standard and emphasize community engagement as a core component [41]. Initiatives such as the Ontario High-Priority Communities Strategy (HPCS) reflect the importance of fostering partnerships with PHAs, FBOs and CBOs to overcome barriers to vaccine acceptance. Generating knowledge about participatory engagement processes is essential to ensure effective intervention design, implementation, and evaluation, as is a thorough understanding of the socio-cultural, political and/or institutional contexts that influence marginalized groups’ trust in vaccines. However, significant research gaps remain regarding the nature of engagement processes used by public health units (PHUs) with FBOs for ethnoracially minoritized populations [42].

Negotiating faith-based partnerships for vaccination are often faced with difficulties due to long-standing, often ideologically based distrust and mistrust between public health and certain religious communities [43]. Improving faith-based partnerships thus requires the generation of knowledge on the contextual factors that promote sustainable engagement processes and the complex dynamics that influence vaccine acceptance among equity seeking groups. In this research, we use “groups made structurally vulnerable”, considering the systemic dynamics that influence their attitudes, beliefs, and mistrust in vaccines. By addressing these gaps, researchers and policymakers can advance evidence-based strategies that effectively enhance vaccine acceptance within hard-to-reach ethnoracial communities. This study therefore aims to explore PHU engagement processes with FBOs. The results can be used to improve the engagement processes and strengthen partnerships that promote equitable public health interventions.

## 2 Methodology

This research is part of a larger pluridiciplinary study that aims to analyze Ontario PHUs’ local partnerships with FBOs to improve vaccine confidence among ethnoracial populations. This qualitative study aims to explore the perceptions, experiences, and collaborative processes of PHUs with FBOs during the COVID-19 vaccine rollout. Specifically, this research will document the processes by which PHUs engaged with FBOs and local communities to build vaccine confidence.

### 2.1 Study setting

The study was conducted in Ontario, Canada’s most populous province which also receives the largest number of immigrants annually. In 2022, Ontario’s population represented around 39% of the total Canadian population. In the fourth quarter of 2022, the province welcomed around 40% of all immigrants to Canada [44]. There are 34 PHUs in Ontario, funded by municipalities and the provincial government [45]. Each PHU covers a specific geographic area and is responsible for protecting and promoting the health of its residents by delivering programs and services. PHUs are headed by local medical officers of health, who are accountable to boards of health.

### 2.2 Analytic approach

We drew on a critical realist ontology [46, 47], which considers the existence of a reality albeit graspable only through the researcher’s interpretation of the perspectives and discourses of research participants who are actors in the experiential context. Language is a channel for accessing information, conceptualized as reflecting the contextual realities of actors [48]. We adopted an experiential epistemological orientation to data interpretation to focus on meaning and significance as attributed by participants. Adopting this approach means that this analysis does not seek to explain the social construction of the partnership between FBOs and PHUs, but rather examines participants’ subjectivity about the construction of the partnership [49]. An experiential orientation was the most appropriate, as it enables us to give priority to PHUs’ own accounts of their attitudes and opinions related to engagement with FBOs, without seeking to analyze the socio-cultural factors that underpinned the development of their attitudes and opinions.

#### 2.2.1 Data collection

34 PHUs were invited by email to participate, and 18 PHUs agreed to an interview. Each PHU interview was conducted via Zoom^™^ with one to three staff members involved in COVID-19 vaccine deployment (i.e., vaccine managers, program managers and medical officers of health). A total of 19 in-depth interviews (two with one PHU), were conducted, each lasting 60–80 minutes. The interviews took place between August and November 2022. Particular attention was given to reserving the anonymity of those interviewed. Participants were asked for free and informed consent after being informed of the pros and cons of participating in the research. Written consent was obtained through an information and consent form signed by each research participant. This project received ethics approval from the University of Toronto (#42490).

A semi-structured guide was used, offering the flexibility to explore participants’ perceptions, experiences, and meanings in depth, while also capturing rich, contextualized data and allowing new avenues of questioning to be pursued. The guide addressed 2 main themes: 1) experiences of partnerships with FBOs, 2) the connection processes and roles and responsibilities of actors. The interviews were conducted by three members of the research team (MS, DB, AK,). MS conducted 15 interviews, DB conducted 3 interviews and observed 12 interviews, and AK acted as an observer for 6 interviews. The team collected documents about PHU-FBO collaborations where available.

#### 2.2.2 Data analysis

The study was not intended to prove or disprove hypotheses or test theories. As such, we adopted a predominantly inductive approach to analysis, prioritizing the meaning of the data based on the participants’ discourse rather than on a predetermined theory or conceptual framework [50]. Thematic analysis (TA) was used to generate the coding framework [51]. More specifically, we adopted an experiential thematic analysis focused on exploring the reality of participants’ contextual experiences, perspectives, and behaviors [52]. This method is a kind of midpoint between coding reliability [53, 54] and the reflexive approach to TA [55]. We followed six steps: 1) data familiarization, 2) systematic data coding and codebook development, 3) initial theme development and codebook consolidation, 4) non-rigid coding guided by the codebook, 5) codebook refinement and main theme development, 6) report writing.

*Data familiarization* was conducted by two team members (KK and AK) who examined the noisy data from several readings, which were then transported into NVivo 12 for coding.

*Systematic data coding and codebook development*. KK and AK first carried out an initial open individual coding of 4 interviews, selected based on PHU size, resource availability, and varying experiences in partnering with FBOs. KK and AK conducted integral coding of each interview based on the interviewees’ discourse [50]. This stage not only allowed us to become more familiar with the data, but also to generate two initial descriptive codebooks.

*Initial theme development and codebook consolidation*. Following input from the core research team, KK and AK merged the two codebooks to generate a hierarchically structured version of the initial themes (theme and sub-themes), which was reviewed by ED. This codebook was used (KK and AK) to code a fifth interview and re-review the first four coded interviews. The team finalized the codebook consolidation process after three iterations, clarifying conceptualizations of themes and sub-themes with input from ED, SF, SA, and AA.

*Non-rigid coding guided by the codebook*. KK conducted a systematic coding of the remaining interviews (n = 15), focusing on the semantic and manifest content of the discourse [48]. The coding enabled us to identify themes and sub-themes related to the processes of engagement of public health units with FBOs, as well as themes related to barriers and facilitators to engagement. In this article, we present only the results on engagement processes.

*Codebook refinement and main theme development*. This stage involved redefining and refining the initial themes and sub-themes based on our analysis, i.e., identifying the essence of each theme and sub-theme [56]. This made it possible to reorganize (moving some content, merging others) to produce a coherent narrative describing the PHUs’ partnership process with FBOs during COVID-19.

## 3. Results: PHU engagement process with faith-based organizations and racialized communities

The results explore the process by which PHUs engaged with FBOs and local communities to build vaccine confidence.

Analysis of the respondents’ discourse enabled us to reconstruct a two-phase process developed by PHUs to engage with faith-based and ethnoracial communities to implement COVID-19 vaccine confidence interventions: an upstream preparatory phase consisting of an internal learning process, sometimes with FBOs about their needs, and the subsequent engagement phase with FBOs (S1 Fig).

### 3.1 Phase 1: Learning process about FBOs and their needs

The upstream learning process consisted of exchange frameworks and use of data by PHUs and to analyze the situation facing groups made structurally vulnerable. Across all PHUs, three decision-making mechanisms/processes emerged from our findings: i)., the use of existing data to better identify and situate groups made structurally vulnerable; ii.) the creation of discussion frameworks to get to know priority communities for engagement to better understand their needs and how to respond effectively; and iii.) internal restructuring to better manage the engagement process.

#### 3.1.1 Use available data to analyze the situation

Most PHUs tried to first understand the situation of equity-seeking ethnoracial and faith-based groups disproportionately affected by COVID-19. They used available data such as socio-demographic and ethnographic profiles of neighborhoods, population heath data, and vaccination records. These data were used to identify priority neighborhoods (i.e., neighborhoods where under-vaccinated communities live), and those not reached by vaccination strategies to modify or adapt the PHU’s vaccination strategy to reach priority groups, and identify areas where partnerships were needed.

*“We have our own*, *it’s called Priority Neighborhoods projects that we have*. *And so*, *we have basically divided our region up into different neighborhoods and we use information from census and from various databases to have a better understanding of the demographics within those different neighborhoods*. *And from there we’re able to extract information that helps us to identify within these different neighborhoods*, *we can look at various demographic information*. *So*, *we were able to track where we were seeing outbreak as well as cases*. *So*, *the number of cases within each of these neighborhoods*. *And then in addition to that we used the information that we had available*, *such as looking at ethnicity*, *what home language was*, *things like that*. *And so from there we were able to prioritize neighborhoods where we wanted to ensure that we were providing clinics”*

Interviwew_1

The use of data also enabled PHUs to better understand the needs of priority groups and identify the best strategy based on their needs. For example, a priority groups’ mistrust in vaccines calls for a different strategy than a priority groups’ issue with vaccine physical accessibility.

*“Some case we knew this was a big hotspot and we knew we did a mass clinic*, *like five-minute drive away*. *And it was still the lowest vaccine coverage in our region*. *So clearly access is not the problem*. *Why are people not coming? Once we better understood the why*, *we were able to better design solutions that could address the why.”*

Interview_9

#### 3.1.2 Reorganize internal structure or mechanism to support engagement with faith-based and ethno-racial organizations

Health equity and system partnership teams already existed in PHUs. These teams were empowered during COVID 19 to engage with FBOs, to set up and facilitate working groups and discussion tables (e.g., vaccine engagement team, priority population planning tables, community planning tables) and to coordinate various action plans (e.g., equity action plan, community coordination plan).

Internal redeployment of staff strengthened the capacity of existing equity teams to support collaborative vaccination efforts. In some cases, PHUs’ communications services were redeployed to support the equity teams.

*We kind of had to consistently restructure in order to adapt from a vaccine delivery perspective*. *We had what I would describe as like key liaison partner leadership that was a mix of communication staff*. *We have a health equity team*, *so our communications health equity team*, *plus components of the operational leadership*, *kind of were working in a shared work model for engaging with community partners and orchestrating those clinics*.

Interview_7

#### 3.1.3 Establish working groups and discussion tables within PHUs

PHUs established working groups and discussion tables to discuss the best ways to engage with faith-based, ethnoracial and community organizations. Three strategies were identified across PHU interviewed:(i) discussions involving only PHU staff, including on occasion other PHUs to share knowledge and experiences; (ii) working groups with health system players (PHU staff, pharmacy, community health centers, primary care clinics, etc.); (iii) intersectoral discussion groups involving PHUs, municipal government, FBOs, and CBOs.

Such exchange mechanisms enabled PHUs to learn more about faith-based and ethnoracial organizations and how to better communicate, reach, involve or engage FBO leaders. PHUs also recognized the importance of working with staff who understood and were sensitive to the cultural context. For example, some spoke the language, or relied on nurses who did home visiting in Amish, dutch reform or the low German speaking Mennonite homes.

Staff meetings and intersectoral discussions were used to decide which priority groups to engage (for example, prioritizing engagement with vaccine hesitant groups, without ignoring vaccine resistant groups), but also to draw on lessons learned from PHUs’ pre-pandemic engagement with faith-based communities to implement immunization strategies. Such discussions enabled PHUs to better understand the context, specific needs, habits, values, and beliefs of groups made structurally vulnerable to target potential FBOs with whom to engage to strategize and optimize engagement practices with priority communities. These exchanges allowed for open dialogue on challenges of engaging with communities, and to identify a range of relevant interventions to support community engagement.

### 3.2 Phase 2: Public health units’ engagement process with religious leaders and faith-based organizations and other CBOs

The majority of PHUs adopted a step-by-step process for engaging with FBOs and ethno-racial communities (i) inform or consult to encourage interaction and open dialogue with FBOs and CBOs; (ii) involve FBOs and CBOs in planning; (iii) collaborate with FBOs, religious leaders, and ethno-racial communities to deliver services. We borrowed terms (consult, involve collaborate) from existing community engagement literature to name the stages we found and reconstructed from our inductive analysis. At each stage, PHUs deployed several intervention strategies to engage with FBOs and ethno-racial communities. These strategies vary according to the FBO’s attitudes about vaccines–hesitant, resistant- and the nature of the relationship–existing or new partnership.

#### 3.2.1 Inform or consult to encourage interaction and open dialogue with FBOs and CBOs

PHUs identified and established contact (“when it didn’t exist”) with religious organizations for early and open dialogue; then tried to maintain this open dialogue with FBOs and umbrella organizations (network or coalitions involving multiple FBOs) throughout the pandemic. The aim was to keep them informed of new developments and address their needs. PHUs also conducted outreach to engage and inform faith communities and CBO leaders to understand and identify their needs. PHUs adapted their strategy depending on whether they had an existing partnership.

- *Establish contacts with religious organizations for early and open dialogue*.

The aim of PHUs was to make themselves known so that they could demonstrate their readiness to support FBOs, umbrella organizations, and faith-based associations (ex; council of imam), mosques, churches, gurdwaras in case of need. To identify potential partners, PHUs used geographic tools (i.e., Forward Sortation Areas) and the Internet to identify FBOs and key common gathering locations. To establish new contacts, PHUs usually deployed two strategies. While one strategy was to “make direct connections where there were no previous relationships”, the other was to “use intermediaries to connect” with FBOs. To establish a new connection, PHUs *used phone*, *email*, *or word of mouth* to connect directly with FBOs (e.g., the Interfaith Council, the mosque, the church, the gurdwara). Most often it was PHU staff who already had links with these FBOs. They also relied on making connections via intermediaries including community ambassadors, community organizations or health system partners to get in touch with FBOs. Community ambassadors were members of ethno-racial communities with a connection to the FBO, who were recruited by PHUs or municipalities. Some PHUs teamed up with their community partners (e.g., newcomer settlement agencies, cultural community learning center, migrant workers’ group) who were already in contact with FBOs to connect with these organizations. Other PHUs worked with health system professionals and partners (community health centers, municipalities, nurses, midwives, physicians) who were already in contact with religious communities to offer health services (maternal and child health, chronic diseases, etc.). They were often relied upon by PHUs to disseminate messages about COVID-19 through public assemblies, press conferences with municipalities, etc.

Other PHUs went through ethno-racial communities to establish links to reach out to groups made structurally vulnerable (e.g., partners from the Black, African, and Caribbean communities or Somali community). For example, some PHUs often go through anti-racism groups or newcomer groups to recruit community leaders. In some cases, ethnoracial communities reached out to collaborate with a PHU. “*It was about six weeks long that we*, *that we trialed that*, *but they were the ones who reached out to us to say*: *we want to do this*, *and this is how we want to do it*.*" And we said*, *"sure*, *we’ll help*.*”* Interview_16

- *Maintain an open dialogue with FBOs*, *keeping them informed of new developments and their concerns and needs*.

After establishing contact, PHUs "kept the door open" with these religious communities, FBOs and umbrella organizations of FBOs to gradually build trust. This involved maintaining links with the organizations or communities with which they were in contact throughout the pandemic, providing them with regular information to keep them abreast of new developments and opportunities to discuss their concerns and needs, while respecting their beliefs.

Building trust required consistent and regular communication with FBOs and religious communities about new vaccine policies and guidelines as they arose. Trust was also maintained through the creation of a feedback mechanism to "listen in" on vaccine-related concerns and identify what was happening on the ground in their communities.

*“we also did proactive messages to faith leaders before notable religious holidays*, *to remind the congregations about being COVID safe*, *thank them for their leadership and supporting and limiting transmission highlighting mask use*, *providing links to OPH signage*, *all of that…… But what we would do is prior to sending those out*, *we would often meet with the faith leaders or have a conversation I should say*, *with the faith leaders to find out like what information they felt was really needed in those messages*, *what would support their community.”*

Intervieew_8

Similarly, PHUs have continued to develop existing relationships with FBOs. In the case of existing partnerships with FBOs and religious communities historically recognized as being very hesitant, most PHUs noted that they respected their values, beliefs, and perspectives.

This approach prevented PHUs from undermining the trust established based on previous collaborative experiences. Regarding new partnerships, some PHUs chose to maintain an open dialogue with hesitant communities in the hope of building trust over time and strengthening the partnership. *And they were certainly one of the partners that we formally named and put effort into connecting with*. *Our leadership wanted to not draw more attention to that group*. *We wanted to continue to lead with kindness and love and say when you’re ready*, *we’re ready*. *When you’re ready to talk to us*, *we’ll be there to talk to you and just continue to take those baby steps as we always do*, *and always have throughout our vaccination journey with many of these communities*. *And so*, *what we try to focus our energy on was not on the complete vaccine opposed*. *We’ll continue to put universal information out there so that people can find us*. *And we won’t question*. *But we put a lot of our*, *our focus on the vaccine hesitant*. Interviwew_12

- *Outreach to engage faith communities and CBO leaders*.

Outreach efforts aimed to establish contact with FBOs, provide them COVID vaccine information and encourage them to collaborate to get vaccine messaging to ethnoracially minoritized populations (e.g., Black, African and Caribbean community; and South Asians). This was one of the strategies for informing and opening a dialogue with FBOs to meet their needs. Early outreach activities with potential or existing partners, led to collaborative actions. This usually consisted of proactive outreach within the community, that is, reaching out to the FBO (e.g., church) or rural community and explicitly asking them to host vaccination clinics and/or to partner with PHUs to promote vaccines. Sometimes proactive outreach consisted of providing these organizations information about the vaccine to share with the communities they serve (e.g., sharing leaflets about where to get vaccinated and what eligibility criteria needs to be met to be vaccinated, public meetings etc.). Nurses and primary care doctors were most often relied upon to address concerns of historically hesitant FBOs and recognized religious leaders with whom a partnership existed. In other cases, PHU staff such as midwives, and nurses conducted home visits.

*We do have good community partnerships with health services that do work with them*. *So*, *we are well connected with the midwife that has connection with them as well*. *So*, *through them and through their questions and answers with them we were able to answer questions that they had as well as through the Inglehart family health team*. *We have a nurse practitioner that’s well immersed within that community as well*, *to try and*, *you know*, *get the information to them*, *and answer their questions*.

Interview 14

#### 3.2.2 Involving faith-based and community organizations in planning

The second step in the engagement process with FBOs consisted of involving FBOs/CBOs in planning and implementation. This involved two steps. They first identified the best way to address community needs according to the characteristics of different faith-based and ethno-racial groups, and then worked together to set-up vaccination clinics.

- *Work with faith-based organizations to identify their needs and plan together how best to support them*.

While some PHUs integrated representatives from FBOs into their existing vaccine working groups, in other cases, it was the FBOs who heard about the working group and reached out. Other PHUs organized discussion sessions–through a working group for instance—with various FBOs and religious leaders outside a formal forum.

Regular exchange meetings between PHUs and FBOs achieved a number of objectives: i) identify the needs of religious leaders (the messages they need for their sermons to promote vaccination; and how frequently as well as the form through which FBOs want to receive this information); ii) identify the needs of the religious communities to address concerns; iii) listen to and discuss with vaccine hesitant communities to design vaccine deployment strategies that conform to their values. iv) ensure through discussions with FBOs that vaccine deployment strategies will be culturally appropriate and gender sensitive.

*So*, *one thing we did early on is we met with the Council of Imams to ask what would best support them*. *And one of the things that they had identified is weekly key messages so that they can use our messages to inform their Friday prayer sermons*. *Highlighting important COVID updates*. *So like socializing*, *vaccine effectiveness*, *providing mental health resources*, *importance of being COVID wise*, *highlighting multilingual resources*, *countering misinformation*, *and what we’re hearing from the community*.

Interview_8

PHUs were often open to learning about FBOs and religious communities to better meet their needs. *They provided education to our teams about their cultural practices about their norms*. *What their community would need from us*, *things that we needed to be conscious of when we were in their space*. *And they were very open and understanding of our learning curves as well in working in a space like that*. Interview_16

- *Obtain permission and involvement of faith-based community leaders to set up vaccination clinics in places of worship*.

This level of engagement led to discussions with FBOs to plan vaccination clinics, such that FBOs agreed either to host clinics in a trusted location of their choice, or to get involved in deploying vaccines as volunteers. PHUs organized meetings with FBOs to address their concerns about vaccines, discuss vaccination clinics and provide information about organizing space for a mobile clinic.

*It was just discussing what would work best for both partners in terms of promotion*, *timing of the clinics*, *staffing that was needed from the health unit to support the clinics*, *volunteers from the mosque itself that would be needed to help support the running of the clinic*. *I would say*, *to put together the plan and who would do what and who could offer*, *what supports (they needed)*

Interview_11

To maintain the confidence of certain partners, who are known to be hesitant, certain PHUs first conducted surveys to ensure that the communities wanted to host these clinics.

*We did do that survey with all the communities; we asked about*, *were we meeting their needs in terms of the information they were receiving*. *And it was also looking for interest in vaccine clinics*, *in their communities*. *So yeah*, *that we didn’t want a heavy-haned approach to COVID vaccines to damage the relationship and then impact uptake in other vaccine programs that we’ve been working so hard to build up*. *In any case in this survey*, *we did find that the one community in particular was willing to host vaccine clinics*, *which I*, *for one wasn’t expecting I don’t know about you guys*, *but we did end up hosting*, *I think two (clinics) there*.

*Interview_5*

PHUs then proceeded to make a coordinated selection of clinic locations, with the involvement of FBOs, and worked with them to set up mobile clinics in places of worship and in their communities. They also linked certain FBOs with the Ministry of Health’s GO-VAXX initiative. This consisted of using a mobile vaccination clinic to administer COVID-19 vaccines without an appointment to people wherever they lived.

In short, this section demonstrates the importance of involving FBOs from needs assessment to planning to implementation. This co-design process considers the needs and specificities of each community.

#### 3.2.3 Collaborate with faith-based organizations, religious leaders, and ethno-racial communities to deliver services

The third stage of the engagement process involved working with FBOs and ethno-racial communities sharing roles and responsibilities for implementing activities. Responsibilities were shared according to each partner’s capabilities. PHUs implemented activities to improve vaccine confidence among groups made structurally vulnerable. Activities included training and informing through public assemblies and webinars, communication campaigns (e.g., poster, video, WhatsApp, Twitter), and setting up vaccination clinics (fixed and mobile). In some cases, PHUs provided financial support to rent suitable vaccination premises to FBOs and CBOs.

These activities were implemented in three ways: (i) work directly with FBOs and ethno-racial communities to inform communities about COVID-19 and related vaccines; (ii) using intermediaries to deliver services, (iii) PHUs worked alone discreetly.

- *Work directly with FBOs and ethno-racial communities to inform communities about Covid and delivering the vaccine*.

Some PHUs engaged directly with FBOs which were usually less hesitant. Some were often the first to reach out and ask for help. These direct collaborations involved working with FBOs to i) adapt communication materials, ii) design communication supports, iii) distribute communication supports, and iv) deliver COVID-19 vaccines.

*Adapting communication materials*. Some tools were developed at the provincial level. religious leaders also contributed by reviewing message content and information (poster, help with image selection, etc.), ensuring its relevance to religious or ethnoracial communities and translating them into plain language (posters and social media messages in multilingual format). For religious communities hesitant or resistant to vaccines, the emphasis was on protection to maintain trust (information on how to protect oneself, washing hands, distancing oneself, staying at home when ill to protect others). The goal was to make messages accessible to different sub-groups (certain resistant or low literacy groups).

*Designing communication materials*. This often involved developing communication media in which leaders expressed their opinion on the vaccine or guides (posters, videos, etc.).

*it involved posters*, *where each one of these leaders…*, *for example*, *they had a quote on the poster about why they got vaccinated*. *It’s not like it’s the same poster slapped with a different person*. *It is an individualized message based on that person’s I guess*, *support intention for the vaccine*. *It was that leader’s personal message to their community about why immunization is important*.

Interview_4

*Communication channels*. FBOs and religious leaders disseminated messages (on the vaccine, and preventative measures such as mask wearing, social distancing, greetings without contact; ministry rules in force, etc,). PHU social media strategies relied on FBOs’ and their leaders’ social media connections (YouTube video, TiKTok, websites, Instagram, Twitter QR code, WhatsApp) to amplify messages (i.e., bulletin boards for poster locations, messages shared by leaders to groups, broadcast on FBOs’ Facebook and Twitter pages). Messages were also disseminated through ethnoracial groups’ social media connections (i.e., Twitter, Facebook, WhatsApp). The ethnoracial groups also provided in-clinic and telephone interpretation services and multilingual radio broadcasting.

*Delivering COVID-19 vaccines*. PHUs worked with FBOs and ethno-racial communities to adapt the vaccine administration strategy. PHU’s delivered vaccines in places where communities felt safe, trusted, familiar or accustomed to frequenting, notably their places of worship, faith-based health centers and ethno-racial community health centers (CHCs).

*Okay*. *So*, *one of the ones that we partnered with most closely would be within our Hindu population*, *locally in the Gurdwara temple*. *They hosted clinics multiple clinics over the course of a year in their space at the temple itself*. *So*, *in the*, *the basement*, *I think it was where they welcomed our team in*. *They provided volunteers*, *they provided food for clients*.

Interview_16

PHUs also mobilized staff who were members of the community to facilitate communication, and places of worship hosting the clinics provided volunteers.

*They also coordinated many of the logistics of the clinics themselves*, *too*. *And suggested the time and day that would work best for their membership*, *which we were able to accommodate just fine*. *So*, *they had volunteers available to help with traffic flow and supporting different aspects of the clinic and then the health unit just offered some of the staffing pieces*, *helping with registration*, *with the vaccination itself*, *the vaccine preparation*. *And there were also members of this organization that were registered healthcare professionals who were nurses*, *doctors*, *pharmacists*, *paramedics*, *and could administer the vaccines themselves*. *we’re doing the vaccination*.

Interview_11

- *PHUs Worked discreetly*.*to respect communities’ need for times for reflection*.

In some cases, PHUs didn’t get the help of FBOs, they respected these religious communities, whose members risked rejection if they were caught vaccinating against COVID-19. They then often organized vaccination clinics outside the community for those who wished to receive the vaccine discreetly.

*It was a really difficult time for the part of their population that wanted to be vaccinated but didn’t feel safe or comfortable to get vaccinated in a spot where they would be seen*. *And so*, *we had to do little mini popup clinics and mobile clinics in adjacent communities*. *And then very clearly advertise them so that they would be able to travel to another community*. *So*, *they wouldn’t be seen getting activated*. *We engaged their primary care providers wherever possible*. *And so*, *we would set up clinics with the primary care providers to help them access the vaccine in a way that it didn’t seem like it was a vaccine clinic*.

Interviwew_17

In some communities, there are divergences between religious leaders towards adopting the vaccine and the government’s orientation to vaccine. PHUs facing these situations did not exploit these differences but had to work discreetly within the community to give those who wished access to the vaccine by setting up mobile clinics to meet the needs of certain groups, specifically hesitant communities.

Some religious communities known to be vaccine hesitant in general, needed a long period of intra-community reflection before the leader committed themself on behalf of the group to encourage vaccination. In some cases, the experience of previous collaborations between certain PHUs and these religious communities has shown that this delay to decide about vaccination can last more than a year. As a result, some PHUs worked with nurses and doctors who are connected to these communities to speak directly to households in these areas. Public health nurses and midwives provided information on COVID-19, the vaccine, answered questions and provided opportunities for the vaccine to be administered directly at home in a discreet manner, based on an individual’s choice.

In some communities that were very hesitant or even resistant to vaccines, PHUs made clinics available, setting them up discreetly without placing too much pressure on these communities to get vaccinated.

*We went to these places*, *not just once we went back repeatedly and we continue to go back repeatedly*. *So*, *it allows people to make that decision*. *“Maybe next time I’ll go” or I heard some people went*, *“I’m gonna go next time.” They had a good experience*, *they were organized*, *they didn’t make me feel isolated”*. *And we continue to build that reputation that will continue to come out and we’ll continue to vaccinate*, *even if it means we get five people we’re still here*.

Interview_12

For these very hesitant groups, PHUs informed the leaders and the community—through the health system’s long-standing collaborators—of vaccination opportunities, passing on information without forcing or pressuring them in any way. PHUs also posted messages in accessible places (e.g., schools, grocery stores, etc.) in their communities.

*So*, *we have kind of three unique Amish communities and each of the bishops have different approaches*, *we did paper print*, *we did have door to door flyers for those communities*, *through our internal staff who knew how to engage the different communities*. *So*, *it’s not a written language*, *it’s a spoken language*. *So*, *print doesn’t help*. *But we did do a healthcare provider newsletter on a weekly basis as well*, *engaging the family health teams that would serve these communities as well*.

Interview_2

- *PHUs used intermediaries for vaccine delivery in faith communities and groups made structurally vulnerable*.

In some cases, PHUs relied on intermediaries to establish contact though community leaders and health system partners to deliver the COVID-19 messages and the vaccine.

Community ambassadors were recruited by PHUs (through municipal or provincial funding) to establish, interact with, and help interpret PHU guidelines for these communities. These people acted as community brokers to FBOs (removing certain barriers, in particular lack of confidence in public health, facilitating access to services, and removing language barriers). The ambassadors made up for staff shortages and made it easier to forge links since the ambassadors come from these communities. The ambassadors played several roles including to identify and contact religious communities and work with religious leaders to design communication materials. The messages behind the campaign were individualized or tailored to address ethical and religious imperative to trust the science behind vaccination. Ambassadors also organized vaccination clinics, meeting with communities to discuss access barriers, taking part in cultural festivals which often have a religious component, helping PHUs identify places/communities for vaccination sites.

*Partners in the health and social services system*. We identified two types of healthcare system partners who played differing roles: those providing outreach care (e.g., community health centers, nurses, ambulance attendants) and those facilitating the availability of service delivery infrastructure such as municipalities, community support organizations, school boards and the private sector. Health professionals most often acted as a channel for directly transmitting messages and vaccines to religious communities. Nurses and doctors working in the communities shared information and administered the vaccine (or often midwives who make home visits in the case of very hesitant communities). Emergency management services (EMS) and emergency medical services (EMS) sometimes provided the vaccine at home. They supported activities involving FBOs in areas close to equity deserving groups. Municipalities provided support by facilitating access to resources and infrastructure for setting up mass or mobile clinics (vaccination sites, public conferences, etc.). Faith-based schools provided information on vaccines particularly to hesitant parents of children in some communities. Support organizations for migrants and newcomers and some private companies facilitated the organization of vaccination clinics on their premises.

## 4 Discussion

This study explored the processes of engagement from the realities of the PHUs’ contextual experiences and perspectives. We found that PHUs have developed a two-phase process for engaging with FBOs and ethnoracial communities. This process involved a wide range of actors. The first phase, referred to as upstream preparation involved an internal learning process. The PHUs created internal frameworks for dialogue that enabled them to discuss and use available data to better understand and learn about the circumstances facing groups made structurally vulnerable and their diverse needs. The second phase involved a three-stage engagement process used to identify, collaborate, and share roles and responsibilities with FBOs and ethno-racial groups to plan and implement vaccine rollout strategies that aimed to improve vaccine confidence and access among these groups.

The findings from this inductive analysis are consistent with the continuum of community engagement described in existing literature [20, 21, 57, 58]. A rapid review identified various collaborative initiatives undertaken by PHUs with FBOs to improve confidence, access, and use of COVID-19 vaccines across Canada among various structurally vulnerable groups [41]. The study identifies several strategies used by PHUs to inform communities (e.g., race-based vaccine education), to consult and involve communities (e.g., race- or religion-based planning and consultation tables), and to collaborate with communities (e.g., community ambassadors, without addressing specifically engagement processes [41]. The strength of our study is that it sheds light on engagement processes that have received less attention in research. Often, studies focus on the evaluation of results [36] without addressing the factors that contributed to the production of these results, i.e., the analysis of upstream processes. These results address the need for contextualized data on participatory engagement processes. By characterizing the engagement processes that contributed to change in communities, these findings can better contribute to the effective replication of interventions. The added value of this research is that it analyzes the community engagement processes with FBOs and CBOs in the specific context of a global health emergency. Community engagement takes time to build trust. But the COVID-19 context is characterized by the implementation of public health measures that make it difficult to establish contact (distancing and containment measures as poor access to FBOs and CBOs due to restrictions on interacting with these communities), and by misinformation that reinforces mistrust in public health services. Apocalyptic speculations about COVID 19 [59], and the antivaccination skepticism of certain religious leaders have often decreased vaccine confidence within certain communities, and hampered vaccination efforts [60]. More generally, committed people of faith may find themselves forced to choose whom to trust—religious leaders or public health experts [43].

The results also show how Ontario’s PHUs have engaged with FBOs in this challenging context, including those historically recognized as hesitant, like Amish communities. One of the specific features of this process was the aims of community engagement [26]. Our results show that certain PHUs were first and foremost preoccupied with addressing equity. In other words, they didn’t aim for an instrumental community partnership, but rather for structural changes that considered people’s needs and expectations. Thus, the use of data during the planning phase made it possible to identify and target structurally marginalized communities to be reached. Song et al. rapid review also show that some Toronto public health teams used a data-driven approach to overlay factors of inequality (race, income, food security, housing, and disability) to identify areas requiring proximity deployment of the COVID-19 vaccine [42]. Beyond the need to build confidence in vaccines and encourage vaccine uptake, the PHUs also wanted to reach these communities, and offer them the opportunity to make choices based on their values and beliefs. So, the involvement of FBOs and CBOs was more than a utilitarian participation [61] to control the pandemic. They tried to deal with mistrust by using empowering, empathetic, non-confrontational, non-coercive and non-judgmental vaccine deployment strategies. Indeed, PHUs have paid attention to the way public health directives are communicated to minimize their perception as conflicting with values such as religious freedom and maximize their perception as consistent with other values, such as service to others. Some PHUs avoided imposing their beliefs and let the FBOs choose the activities that suited them best. In addition, they did not insist, when the FBOs they wanted to work with, were not in favor of COVID-19 vaccines. Similarly, PHUs respected the collective beliefs of FBOs by not exploiting divergent positions within a community, but rather by exercising discretion and creating suitable spaces for those who wanted to receive the vaccines. This also involved being consistent in messages addressed to specific groups (e.g., migrant workers), recognizing that hesitation is normal, and that the vaccination site is also a place to get answers to questions (which can help to build trust). PHUs mentioned did not pressure the community to get vaccinated while showing willingness to support and respond to community needs. This attitude may have contributed to establishing trust with existing and newly established partners. As with Ontario PHUs, in the US, the Association of State and Territorial Health Officials (ASTHO) partnered with FBOs to reach equity-seeking populations [32]. ASTHO, FBOs and community organizations are interested in working together. Most often, ASTHO has turned to FBOs for help to promote COVID-19 vaccination, but FBOs and CBOs have also turned first to PHUs [32]. To build and maintain trust, it is essential to establish ongoing relationships between structurally marginalized groups and public health organizations, based on mutual respect, reciprocal learning, and a sense of belonging [62]. A deep commitment on the part of immunization program implementers and influential, respected people in the community, such as religious organizations, is paramount. The knowledge generated can be used to improve the design and implementation of equity-focused partnership interventions to build the confidence of structurally vulnerable groups in vaccines.

However, our study has limitations in that it only reports on the experiences of those involved in public health units. FBOs as participants may have provided different perspectives of the engagement process. Nevertheless, the results have implications for public health practice.

The PHUs have privileged a co-design process to consider the needs and specificities of each community during the needs assessment and planning phases, and a co-leadership process for implementation. Engaging with religious communities is a process that must be built over time and requires a great deal of time and patience. Even in religious communities that are hesitant or resistant to vaccines, partnership is necessary. Partnership over time creates clear boundaries and clear expectations of what they are willing to do and what they are unwilling to do. When the partnership exists and is formalized, the players know each other, and the PHUs know what is permitted and accepted by the FBOs. So, community engagement based on equity, mutual respect for values and beliefs, are necessary to reduce mistrust of vaccines and the healthcare system. In this way, making engagement with FBOs a priority strategy and devoting substantial resources (human, financial, and duration) is necessary to improve vaccine confidence among ethno-racial groups seeking equity.

## 5 Conclusion

In Ontario, collaborations between the PHUs and FBOs and other community organizations were initiated to implement interventions aimed at building vaccine confidence among ethnoracial communities. The results of this study shed light on the processes of engagement in the unusual context of the COVID-19 pandemic and contending with distrust in the health system and misinformation about the virus and the vaccine. The COVID-19 pandemic highlighted vaccine hesitancy among ethno-racial and minority populations as a major obstacle to equity. These populations have historically low confidence in vaccines [3] due to a legacy of systemic racism and lack of trust in public health organizations [10]. The PHUs adopted an equity-focused approach, recognizing the weight of structural inequalities. They created internal frameworks for dialogue that enabled discussion and use of available data to better understand the situation of these equity-seeking groups and their diverse needs. They initiated open and early interactions and dialogues with FBOs and ethno-racial groups that helped identify potential partners, establish contacts (where they didn’t exist), work with FBOs and interested communities to plan and implement strategies aimed at deploying and improving trust in vaccines. The results show that PHUs tried to establish new partnerships and strengthen existing ones, to build trust in vaccines while respecting the beliefs and values of FBOs. PHUs’ openness to honest discussion with FBOs, relationships based on respect for different beliefs and opinions on vaccines, and previous experience of working together facilitated the engagement process. Future research into the engagement process with historically hesitant communities will also be needed to document their experiences and perceptions of the engagement process with PHUs. This will provide insights from the perspectives of different stakeholders and contribute to policy and practice decisions to improve equity for groups made structurally vulnerable.

## Supporting information

S1 Fig This is S1 Fig, an appendix A, PHUs engagement process with FBOs. (PPTX)
