# Supplementary material for: How have Ontario Public Health units engaged with faith-based organizations to build confidence in COVID-19 vaccines among ethno-racial communities
Source: PLOS Glob Public Health. 2024 Dec 31;4(12):e0003924. doi: 10.1371/journal.pgph.0003924 (PMC11687800; doi:10.1371/journal.pgph.0003924)
Supplement: S1 Fig — (PPTX) [file pgph.0003924.s001.pptx]

## Slide 1
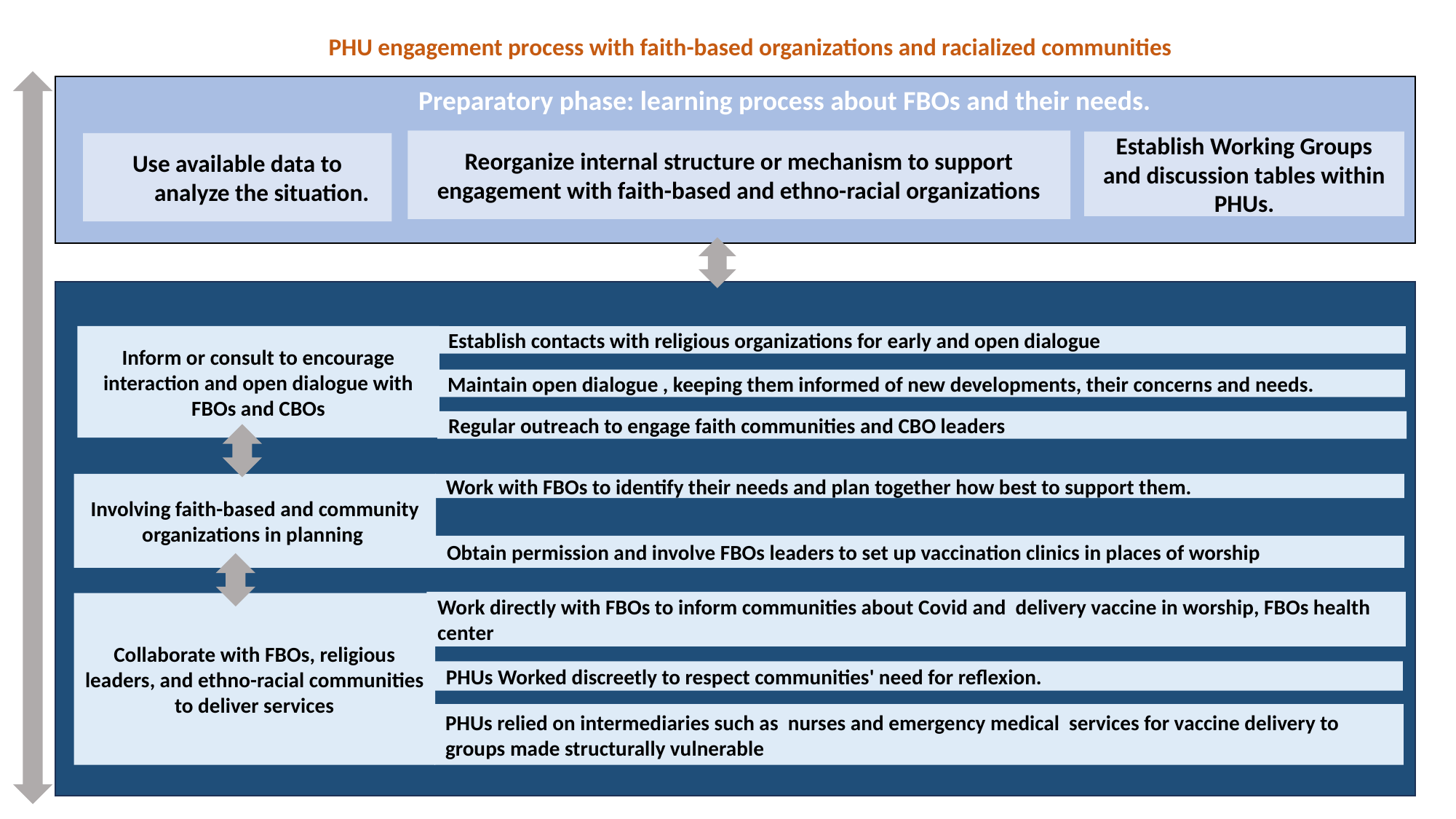

PHU engagement process with faith-based organizations and racialized communities
Preparatory phase: learning process about FBOs and their needs.
Reorganize internal structure or mechanism to support engagement with faith-based and ethno-racial organizations
Establish Working Groups and discussion tables within PHUs.
Use available data to analyze the situation.
Inform or consult to encourage interaction and open dialogue with FBOs and CBOs
Establish contacts with religious organizations for early and open dialogue
Maintain open dialogue , keeping them informed of new developments, their concerns and needs.
Regular outreach to engage faith communities and CBO leaders
Work with FBOs to identify their needs and plan together how best to support them.
Involving faith-based and community organizations in planning
Obtain permission and involve FBOs leaders to set up vaccination clinics in places of worship
Work directly with FBOs to inform communities about Covid and delivery vaccine in worship, FBOs health center
Collaborate with FBOs, religious leaders, and ethno-racial communities to deliver services
PHUs Worked discreetly to respect communities' need for reflexion.
PHUs relied on intermediaries such as nurses and emergency medical services for vaccine delivery to groups made structurally vulnerable
